# National Consensus for the Management of Acute Gastroenteritis in Jordanian Children: Consensus Recommendations Endorsed by the Jordanian Paediatric Society

**DOI:** 10.1155/2022/4456232

**Published:** 2022-08-30

**Authors:** Mohammed Rawashdeh, Basim Al-Zoubi, Maha Barbar Aliwat, Salma Burayzat, Esam Alhindawi, Ali Attia Al-Matti, Eyad Altamimi

**Affiliations:** ^1^Jordan University and Hospital, Amman, Jordan; ^2^Prince Hamzah Hospital and AlBasheer Hospital, Amman, Jordan; ^3^King Hussein Cancer Centre, Amman, Jordan; ^4^Department of Pediatrics, Faculty of Medicine, The Hashemite University, P.O. Box 330127, Zarqa 13133, Jordan; ^5^Private Sector, Amman, Jordan; ^6^Paediatric Department, Istiklal Hospital, Amman, Jordan; ^7^Jordan University of Science and Technology, Irbid, Jordan

## Abstract

Diarrhoeal diseases are one of the leading worldwide preventable causes of death among children under 5 years of age. Almost half of children do not receive optimal acute gastroenteritis (AGE) treatment in Jordan. With neither regional nor local guidelines available for AGE, consensus recommendations on the management of paediatric AGE in Jordan were developed by a panel of senior paediatricians and paediatric gastroenterologists and are endorsed by the Jordanian Paediatric Society. Recommendations are based on international guidelines and available relevant literature in relation to the AGE landscape and the healthcare system in Jordan. The prevention of diarrhoeal diseases should focus on the improvement of nutrition, hygiene, and sanitation, the introduction of routine vaccination against rotavirus, and the adoption of a standardised approach for AGE management (oral rehydration solution (ORS) use±adjunct therapies, continued feeding, and avoiding routine antibiotic use). Ondansetron, diosmectite, racecadotril, probiotics, and zinc can be considered adjunct to ORS, if needed. Local data gaps should be addressed. The clinical algorithm for the management of paediatric AGE could promote adherence to practice recommendations and by extension improve health outcomes in children.

## 1. Background

Diarrhoeal diseases are one of the leading worldwide preventable causes of death among children under 5 years of age [1]. In 2017, diarrhoeal diseases in children 5 years and younger were responsible for 440,521 deaths globally [2]. Rotavirus was the most common cause of severe diarrhoea in children [3] and is therefore an important contributor to morbidity, mortality, and hospitalisation among children under 5 years [4]. In the Middle East, up to 78% of hospitalised acute gastroenteritis (AGE) cases were attributable to rotavirus [4]. Despite the high rate of bacterial and viral gastroenteritis in Jordan [5–9], almost half of children do not receive optimal AGE treatment [10]. Rotavirus vaccination was introduced in 2015 to the national immunisation program and seemingly proved effective in reducing rotavirus-related gastroenteritis [11]. Despite this, child mortality due to diarrhoeal diseases is still reported, with 3% of deaths among Jordanian children under 5 years in 2017 attributed to diarrhoea [2]. The Federation of International Societies of Paediatric Gastroenterology, Hepatology and Nutrition (FISPGHAN) has prioritised universal rotavirus immunisation, early use of oral rehydration solution (ORS), and the limitation of inappropriate medical interventions when addressing paediatric AGE [12]. With neither regional nor local guidelines available for AGE, a consensus was reached on the management of paediatric AGE in Jordan. Consensus statements were provided that adapt international recommendations on AGE management to the national context of Jordan and its healthcare system. Consensus recommendations are endorsed by the Jordanian Paediatric Society.

## 2. Methodology

A panel of senior paediatricians and paediatric gastroenterologists reviewed international guidelines and available relevant literature in relation to the AGE landscape and the healthcare system in Jordan. Statements on the diagnosis, clinical management, and prevention of AGE were suggested and discussed in an initial meeting. Draft statements were then developed, adopted or adapted from international guidelines, and voted upon in a second meeting. The final statement wording was deliberated by the panel and established based on a majority voting system (≥70% agreement). Rehydration and pharmacological therapy were evidence-based and consistent with international guidelines. Where applicable, recommendations were adjusted to reflect local availability and local labels. The national consensus on the management of paediatric AGE in Jordan was then drafted and reviewed by all authors.

## 3. Definition of AGE

To date, there is no universal definition for AGE. However, the 2014 joint European guidelines for the management of AGE in children is widely accepted in clinical practice; also, studies and its definition of AGE were therefore adapted in Jordan. It was found appropriate to omit the number of typical evacuations per day as a criterion for AGE to avoid AGE overdiagnosis since stool frequency varies by age and diet [13, 14]. High bowel frequency is not uncommon in the first month of life, in which period infants can evacuate more than 6 times per day [13]. This omission also serves to further emphasise the importance of considering stool consistency rather than stool frequency in the diagnosis of AGE. Persistent diarrhoea was beyond the scope of this consensus, which focuses on the management of acute diarrhoea in both infants and children. The concept of persistent diarrhoea is well addressed in international guidelines, such as the European Society for Paediatric Gastroenterology Hepatology and Nutrition/European Society for Paediatric Infectious Diseases (ESPGHAN/ESPID) 2014 guidelines [15]. Consequently, the following simplified definition of AGE was agreed on as follows.


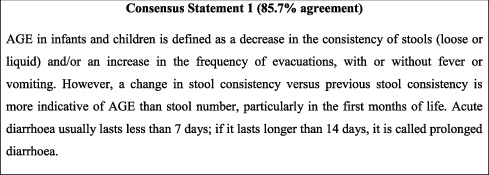


## 4. Burden of AGE in Jordan

While few and widely dispersed across the years, epidemiological studies have attempted to paint the AGE landscape in Jordan. Rotavirus was shown to be a persistently predominant cause of AGE in Jordanian children across the years [5–9], accounting for almost half of all AGE cases in 2011 [16]. Despite continued local efforts and rotavirus vaccination campaigns, diarrhoea-related mortality, morbidity, and hospital admission remain high in Jordan due to the presence of millions of refugees in the country, the pandemic of the coronavirus disease 2019 (COVID-19), the worsening economic status, and the poor utilisation of medical services. In total, 3% of deaths among Jordanian children under 5 years of age were attributed to diarrhoea in 2017 [2], and poor household conditions play a major role in the occurrence of diarrhoea in low-income/rural settings [17, 18]. Maternal education and inadequate home-based AGE management practices are also prevalent [18, 19], which further contribute to the burden of this disease among children. Further nationwide studies and public health campaigns are needed in order to promote AGE-related awareness and establish the impact of improved sanitation, rotavirus immunisation, and the burden of refugees on the epidemiology, severity, and aetiology of diarrhoea among paediatric populations in Jordan.


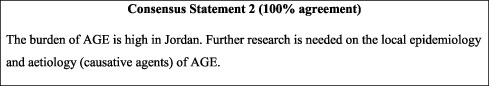


## 5. Prevention of AGE

The burden of rotavirus-related gastroenteritis is evident on economic level (healthcare cost) and healthcare utilisation (primary care visits, emergency department visits, and hospitalization) in countries with no routine rotavirus immunisation [20, 21], even in high-income settings [22]. As such, the benefit of the introduction of rotavirus vaccines into national immunisation programs has been clear-cut in regard to the prevention of rotavirus diarrhoeal episodes, particularly in high-risk countries with high morbidity and mortality [23, 24]. The introduction of the rotavirus vaccine in Jordan in 2015 led to a notable decrease in rotavirus-related gastroenteritis from approximately 50% [16] to 6% [11] of paediatric AGE cases. The high rotavirus vaccination rate (90 to 95%) reported in Jordan is in line with the FISPGHAN priorities in AGE management [12]. However, rotavirus vaccination alone is not sufficient to address the burden of AGE. Other common viral and bacterial aetiologies that are expected to increase in prevalence after comprehensive rotavirus vaccination should also be included in public health priorities. Additionally, transient humanitarian/global emergencies such as the COVID-19 pandemic and refugees should be accounted for considering their influence on the AGE landscape. Poor nutrition and unsafe water/sanitation are the leading risk factors of diarrhoea-related mortality [25] and were significantly associated with diarrhoeal episodes among children in Jordan [17]. Thus, it is essential to provide health education promoting the importance of good sanitation and nutrition (i.e., hand washing, access to clean water, and access to clean toilets), particularly among high-risk low-resource settings (e.g., refugee camps [8] and rural areas [17]) within Jordan.


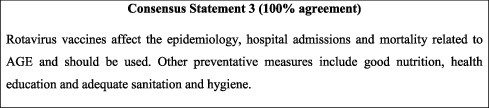


## 6. Dehydration Assessment

Dehydration is one of the major consequences of diarrhoea and has long been used to assess its extent and severity. While generally impracticable, dehydration can most accurately be assessed through percentage body weight lost. Alternatively, the FISPGHAN universal recommendations for AGE suggest the use of physical parameters such as skin turgor, sunken eyes, general appearance, capillary refill time, and mucous membranes for the assessment of dehydration [26]. In this spirit, the dehydration scales incorporating these criteria can be used in practice, the most reliable of which is the Clinical Dehydration Scale (CDS) (Table 1) [27]. The CDS is a validated tool which assesses dehydration based on four clinical characteristics, namely, general appearance, eyes, mucous membranes, and tears. The scoring system allows physicians to classify patients as having no dehydration, some dehydration, or moderate-to-severe dehydration [28, 29]. However, some studies suggest that collective evidence reveals the limitations of the diagnostic value of CDS, particularly in low-income settings [30–32]. That being said, data in this regard remain limited. When possible, children presenting with AGE should undergo complete physical evaluation and the CDS can be used in low-resource, outpatient, or primary care settings where necessary physical parameters cannot be assessed.


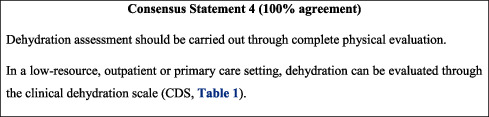


## 7. Diagnostic Workup

Both the ESPGHAN/ESPID and more recent FISPGHAN recommendations emphasise the dispensability of diagnostic workup for the vast majority of children with AGE. Exceptions include cases where complications might arise and be life-threatening, such as symptoms persisting for more than 7 days, extremely severe clinical presentation (e.g., sepsis), travel to high-risk areas or during disease outbreaks, and patients who have underlying chronic conditions that render them susceptible to infections (e.g., cancer and immune deficiency). Consistently, these criteria are adopted in the current consensus, with the addition of an age cut-off of 6 months. Viral AGE most often occurs in younger age groups [33, 34], who are more susceptible to diseases. Moreover, children below the age of 6 months often benefit through breastfeeding from the protective effect of maternal antibodies, which also protect against viral pathogens [35]. The neonatal and early infancy period is characterised by limited/immature immune defences and consequently reliance on maternal immunity and high susceptibility to infections [36]. Diagnostic investigations should therefore be strongly considered in children aged 6 months or younger in order to adequately address any possibly severe infection that might be manifesting as AGE. To note that clinical judgment is essential in determining the need for diagnostic investigations, this should not be abused. This is particularly important in Jordan, where conducting diagnostic investigations for all children below the age of 6 months presenting with AGE might not be feasible in high-volume centres. Moreover, some centres in low-resource regions do not have the capacity to conduct simple bloodwork, and as such, this recommendation would not be applicable across all Jordan.


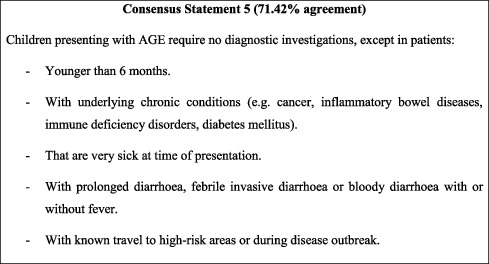


## 8. Indications for Hospital Admission

In case of AGE, physicians should know when to admit patients to the hospital. This not only would allow providing adequate medical care for critical or high-risk cases but also would serve to reduce the burden on the already-struggling healthcare system through the prevention of overadmission. The indications for hospital admission set forth by the ESPGHAN/ESPID 2014 guidelines were therefore adopted in this consensus, with the addition of cases presenting with electrolyte and/or acid/base imbalance. The latter serves to emphasise the importance of correcting imbalances in the dehydrated child and of preventing progression to serious clinical complications. Accordingly, it is recommended that high-risk patients be admitted and closely monitored until response to treatment and improvement. Discharge criteria are left to the clinical judgment of the treating physician.


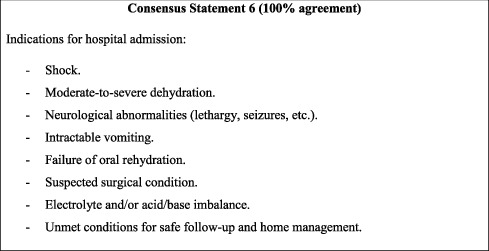


## 9. Complications of AGE

Many clinical complications are associated with AGE in children, which could lead to serious and severe outcomes. If untreated, AGE can lead to clinical deterioration manifesting as acute kidney injury, metabolic acidosis, neurologic manifestations, and encephalopathy [37–39], among other serious complications in addition to severe dehydration-related shock and death [40]. Early intervention and prophylaxis are therefore essential for the prevention of AGE symptom progression and poor clinical outcomes, particularly among high-risk paediatric populations [41].


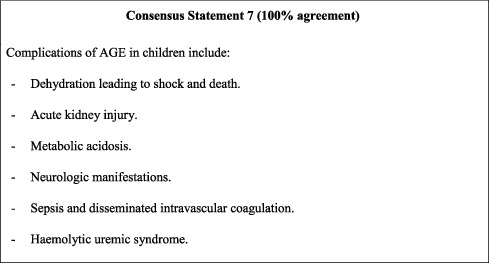


## 10. Management of AGE

### 10.1. Rehydration

Rehydration remains the cornerstone of AGE treatment. The majority of available clinical guidelines agree on the use of ORS for the first-line management of mild to moderate dehydration and intravenous rehydration in case of severe dehydration [42]. Recent years have seen a general trend towards the recommendation and use of reduced osmolarity solutions (60–75 mmol Na+) for the rehydration of children with mild to moderate dehydration who can tolerate oral solutions [26]. ORS was found to be comparable to intravenous (IV) rehydration for moderately dehydrated patients, which can thus spare the risks and increased hospital stay associated with IV therapy [43, 44]. Different ORS compositions are available with wide geographical variability. In Jordan, ORS options remain limited to reduced osmolarity World Health Organization (WHO) formulation solutions (75 mmol Na+). In the case of severe dehydration or failure of oral rehydration, IV rehydration should be attempted when possible. Rehydration through a nasogastric tube is an effective and safe way to deliver rehydration fluid [45] that can be attempted if an IV line cannot be established. The recommendations of the FISPGHAN Working Group were adopted in the present consensus with minor modifications taking into consideration available ORS concentrations [26]. Meta-analyses of available evidence show little to no differences between rapid (1-2 hours) and slow (2-4 hours) IV rehydration among stable children in terms of treatment failure and rehydration rates [46, 47]. However, rapid rehydration might lead to longer hospitalisation and higher risk of readmission [48]. This is in line with the use of IV rehydration, which generally carries higher risks compared to oral rehydration therapy [44].


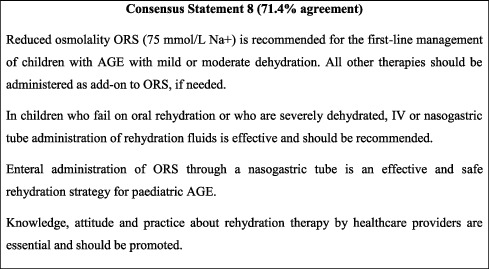


### 10.2. Nutritional Management

In line with available evidence [49], the vast majority of guidelines support early refeeding and recommend against food discontinuation or diet modification [15, 26, 42]. However, the concept of early refeeding of children with diarrhoea remains poorly practiced in Jordan. United Nations Children's Fund (UNICEF) data shows that in 2018, only 46.2% of children under 5 years of age were receiving oral rehydration and continued feeding as treatment for AGE [10]. As such, health education is needed to promote good management practices for acute diarrhoea. Families should be counselled on the importance of early reestablishment of prediarrhoeal diet and should be warned against the use of traditional home remedies such as liquids with high sugar content (i.e., juices), diluted formulas, and carbonated drinks. Refeeding (prediarrhoeal diet) should be attempted 4 to 6 hours after initiation of rehydration therapy. This is not applicable to breastfeeding, which should continue throughout rehydration therapy. Switching to a lactose-free diet is generally not recommended but could be beneficial for the reduction of treatment failure and time till resolution of diarrhoea [50, 51]. However, available data are of low quality and none are available from low-income countries such as Jordan, and lactose-free diets should be reserved for persistent AGE and hospitalised formula-fed children.


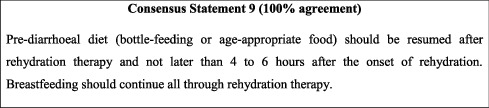



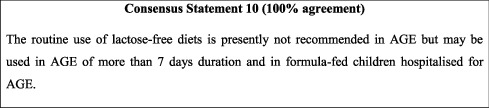



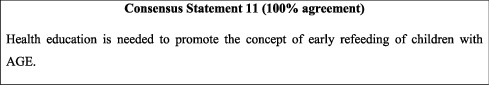


### 10.3. Pharmacological Management

#### 10.3.1. Motility Inhibitors

Loperamide was the only intervention that proved to carry a higher risk when compared to standard therapy among those examined for efficacy in the reduction of diarrhoea duration in children [52]. Loperamide is not approved for use in children aged 12 years or below. There is no strong clinical evidence supporting the effectiveness of loperamide and other antimotility drugs, such as diphenoxylate/atropine, for the management of paediatric AGE. In line with international guidelines, motility inhibitors are not recommended for active AGE treatment [15, 26]. Loperamide should be especially avoided for serious cases of AGE (i.e., associated with infection and/or bloody diarrhoea) [53].


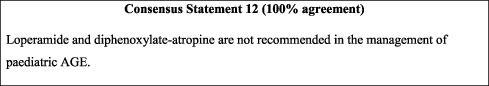


#### 10.3.2. Antiemetics

Seeing as the primary goal of AGE management is the prevention/correction of dehydration, the use of antiemetics could be considered in children older than 6 months of age for the reduction of dehydration due to vomiting. Available studies support the use of a single dose of ondansetron in paediatric AGE and report its efficacy in reducing the failure of oral rehydration therapy and the subsequent need for IV rehydration, in addition to reducing hospitalisation rates [54]. While evidence remains of relatively low quality, ondansetron was the only antiemetic that proved safe and effective in paediatric AGE [55] and is the only antiemetic recommended or considered in international guidelines [15, 26]. That being said, both the cost and the side effects (QT prolongation, severe cardiac arrhythmias) of ondansetron should be carefully considered prior to its use in this setting.


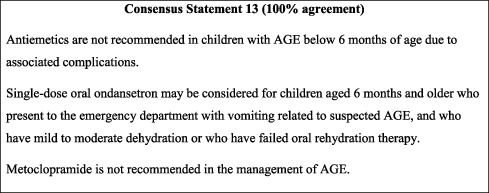


#### 10.3.3. Adsorbents

Other absorbents (kaolin-pectin and attapulgite-activated charcoal) are not recommended.

Studies suggest that adjuvant smectite may reduce the duration of AGE, speed recovery, and reduce stool output [56]. Smectite in combination with zinc could be an effective intervention in the treatment of diarrhoea in children [52]. Smectite is the only adsorbent recommended in international guidelines [15, 26, 42] and was also adopted in this consensus for children aged 2 years and older. This is in line with the recommendation of the French National Agency for the Safety of Medicines and Health Products against the use of clay-based medicine in children below 2 years of age due to possible lead poisoning [57].


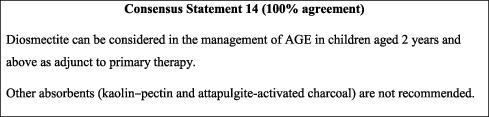


#### 10.3.4. Anti-Infective Therapy

Considering that AGE in children is generally a self-limiting condition, anti-infective therapy is not needed routinely for otherwise healthy children. The ESPGHAN/ESPID pathogen- and setting-based approach for infective therapy [15] was deemed appropriate and adopted in the present consensus (see Table 2 and consensus statement 15). Recommended drug of choice and dosages are subject to change at the treating physician's discretion in accordance with local regulations.


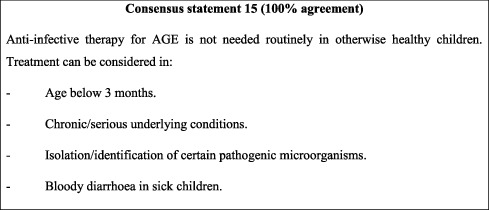


#### 10.3.5. Antisecretory Therapy

Available evidence shows that racecadotril can be safely used in both adults, children, and infants aged 1 month and older. Data support the tolerability and efficacy of racecadotril among children on different outcomes, including the reduction of time to AGE cure in both outpatient and hospital settings [58], ensuring fewer repeated visits to the emergency department before recovery from a diarrhoeal episode [59], and the clinical reduction of diarrhoea in terms of stool output, stool number, and diarrhoea duration [60, 61], regardless of baseline conditions (level of dehydration, rotavirus status, or age), treatment conditions, or cultural environment [61]. Racecadotril in children could also help in limiting the failure of rehydration therapy [62], and the drug has been shown to be more efficacious than other treatments (e.g., probiotics and smectite), with a higher tolerability than loperamide [58]. Its use in paediatric AGE was supported by the ESPGHAN/ESPID guidelines [15] and several others [42] as adjunct to ORS.


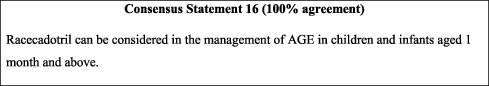


#### 10.3.6. Probiotics

The efficacy of probiotics for the treatment of diarrhoea in the general population remains debatable when symptoms last longer than 48 hours [63]. Some studies show no benefit of *Lactobacillus rhamnosus* GG on the outcomes of children regardless of age, weight, and dose administered when compared to placebo [64, 65]. Routine probiotic administration is also not supported irrespective of the viral pathogen responsible for AGE [66], and the lack of efficacy as a treatment persists even if two probiotic strains are combined [67]. Another study also showed that *L. reuteri* administration cannot prevent the occurrence of diarrhoea in children [68]. That being said, the addition of a probiotic (*L. reuteri*) to standard rehydration therapy significantly reduced duration of hospitalisation of children with AGE, while not affecting duration of symptoms [69]. As such, while probiotics alone might not be effective as AGE treatment or prophylaxis, their use as add-on therapy to standard rehydration ensures an improvement in diarrhoea frequency and duration of the condition as well as hospital stay [70, 71] and is recommended in this setting (≥10^10^ CFU/day for *Lactobacillus rhamnosus* GG and 250-750 mg/day for *Saccharomyces boulardii*) [72].


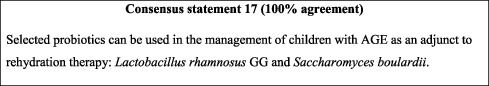


#### 10.3.7. Zinc

Available evidence supports the use of zinc in children aged 6 months or older should they be malnourished or be at a high-risk of zinc deficiency. In these settings, oral zinc supplementation helps by reducing the duration of symptoms, thereby preventing persistent diarrhoea in this age group, with no effect in children younger than 6 months [73]. The addition of zinc to ORS has proven effective in the reduction of diarrhoea severity and duration among children [74]. The addition of zinc alone or concomitantly with probiotics (*Saccharomyces boulardii*) or smectite seems to be one of the most effective strategies for the reduction of diarrhoea duration in children [52]. The enrichment of ORS with both zinc and probiotics was also shown to be beneficial in well-nourished, nonhospitalised infants [75]. Based on this and considering the suboptimal zinc intake in the Eastern Mediterranean region [76, 77] and the potentially high rate of zinc deficiency among Jordanian children [78], zinc supplementation is recommended as add-on to ORS when possible.


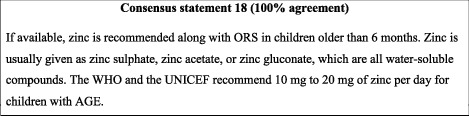


## 11. AGE Treatment Algorithm

**Figure 1:**
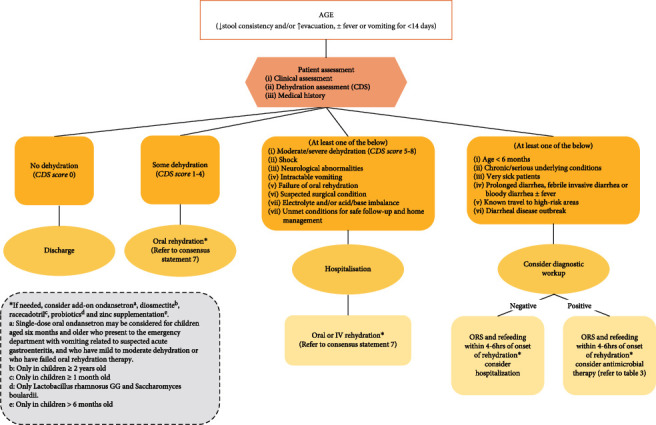
Treatment algorithm for paediatric AGE in Jordan. AGE: acute gastroenteritis; CDS: Clinical Dehydration Scale; IV: intravenous; ORS: oral rehydration solution.

This consensus guideline provided recommendations for the management of paediatric AGE in Jordan, which could be summarised in the following proposed treatment algorithm (Figure 1). Pharmacological treatment options are summarised in Table 3, while antimicrobial treatment options are provided in Table 2.

## 12. Conclusions

The burden of AGE remains notable in Jordan. A national consensus on paediatric AGE management might help alleviate the suboptimal adherence to international guidelines and recommended AGE management and by extension improve AGE outcomes in children. The prevention of diarrhoeal diseases should focus on the improvement of nutrition, hygiene, and sanitation, the introduction of routine vaccination against rotavirus, and the adoption of a standardised approach for AGE management (ORS use±adjunct therapies, continued feeding, and avoiding routine antibiotic use). Local data gaps should be addressed in order to better adapt international recommendations to the particular needs of Jordan, its population, and its healthcare system.

## Figures and Tables

**Table 1:** The Clinical Dehydration Scale (CDS).

Characteristic	Score		
	0	1	2
General appearance	Normal	Thirsty, restless, or lethargic, but irritable when touched	Drowsy, limp, cold, or sweaty; ±comatose
Eyes	Normal	Slightly sunken	Very sunken
Mucous membranes	Moist	Sticky	Dry
Tears	Tears	Decreased tears	Absent tears

Score 0: no dehydration; scores 1-4: some dehydration; scores 5-8: moderate/severe dehydration. Table reprinted with permission from Friedman et al. [29].

**Table 2:** Antimicrobial therapy for infective gastroenteritis.

Pathogen	Indication for antibiotic therapy	Drug of choice^∗^	Alternative agents
*Shigella* spp.	Proven or suspected shigellosis	Oral: azithromycin (12 mg/kg on day 1, followed by 6 mg/kg for 4 days); parenteral, IV, IM: ceftriaxone (50–mg/kg for 2-5 days)	Cefixime (8 mg/kg per day); ciprofloxacin^z^ PO (20-30 mg/kg per day). For a known susceptible strain: TMP/SMX^y^ (8 mg/kg per day of TMP) or ampicillin (100 mg/kg per day) or nalidixic acid (55 mg/kg per day)
*Salmonella* spp. (nontyphoidal)	Antibiotic therapy is indicated only in high-risk children to reduce the risk of bacteraemia and extraintestinal focal infections	Ceftriaxone (50–100 mg/kg per day)	Azithromycin (10 mg/kg per day); ciprofloxacin^z^ PO (20-30 mg/kg per day); for a known susceptible strain, TMP/SMX (8 mg/kg day of TMP)
*Campylobacter* spp.	Antibiotic therapy is recommended mainly for the dysenteric Campylobacter gastroenteritis and most efficacious when started within 3 days after the onset of the disease	Azithromycin (10 mg/kg per day for 3 days or a single dose of 30 mg/kg)	Doxycycline (>8 years) or ciprofloxacin (>17 years), when susceptible
*Shiga* toxin-producing *Escherichia coli*	Antibiotic therapy is not recommended	—	—
Enterotoxigenic; *Escherichia coli*	Antibiotic therapy is recommended, mainly for a traveller's diarrhoea	Azithromycin (10 mg/kg per day for 3 days)	Cefixime (8 mg/kg per day for 5 days); TMP/SMX (8 mg/kg per day of TMP); ciprofloxacin PO (20–30 mg/kg per day); rifaximin (>12 years, 600 mg/day, for 3 days)
*Vibrio cholerae*	Antibiotic therapy is recommended for confirmed or suspected case by travel history	Azithromycin (10 mg/kg per day for 3 days or a single 20 mg/kg dose)	Doxycycline (>8 years), ciprofloxacin (>17 years), or TMP/SMX (when susceptible)
*Clostridium difficile*	Antibiotic therapy is recommended for moderate and severe cases	Metronidazole (30 mg/kg per day for 10 days)	Vancomycin PO (40 mg/kg per day)

PO: per os (by mouth). ∗ depends on a local antibiotic susceptibility profile, which should be monitored. ^y^TMP/SMX: trimethoprim-sulfamethoxazole. ^z^Ciprofloxacin is usually not recommended in the paediatric age group, but it can be used in children < 17 years when an alternative is not feasible. Table reprinted with permission from Guarino et al. [15]. Drug dosage is subject to change according to local regulations at the discretion of the treating physician. For more details, refer to the relevant section in Guarino et al.'s [15] guidelines.

**Table 3:** Pharmacological options as adjunct treatment to ORS for the management of paediatric AGE in Jordan.

Type	Dosage	Time	Notes
Ondansetron	4 mg	Single dose	Six months and older Suspected AGE Failed oral rehydration therapy
Diosmectite	4 sachets (3 g each) a day for 3 days, then 2 sachets (3 g each) per day	5 days	2 years and older
Racecadotril	1.5 mg/kg per administration; 3 times per day (plus one initial dose on day 1) (i) From 1 month to 9 months (less than 9 kg): 1 sachet (10 mg) per administration (ii) From 9 to 30 months (about 9 to 13 kg): 2 sachets per administration	Up to 7 days	1 month and older
Anti-infective drugs	Pathogen-based, see Table 2	Pathogen-based, see Table 2	Consider in (i) Age below 3 months (ii) Chronic/serious underlying conditions (i) Isolation/identification of certain pathogenic microorganisms (ii) Bloody diarrhoea in sick children
Lactobacillus rhamnosus GG	≥10^10^ CFU/day	5-7 days	All children
Saccharomyces boulardii	250-750 mg/day	5-7 days	All children
Zinc	10-20 mg	10-14 days	Use in children older than 6 months

## Data Availability

No data were used to support this study.

## Abbreviations

AGE: Acute gastroenteritis
CDS: Clinical Dehydration Scale
COVID-19: Coronavirus disease 2019
ESPGHAN: European Society for Paediatric Gastroenterology Hepatology and Nutrition
ESPID: European Society for Paediatric Infectious Diseases
FISPGHAN: Federation of International Societies of Paediatric Gastroenterology, Hepatology and Nutrition
IV: Intravenous
ORS: Oral rehydration solution
PO: Per os
TMP/SMX: Trimethoprim-sulfamethoxazole.
UNICEF: United Nations Children's Fund
WHO: World Health Organization.
